# Neurons containing orexin or melanin concentrating hormone reciprocally regulate wake and sleep

**DOI:** 10.3389/fnsys.2014.00244

**Published:** 2015-01-08

**Authors:** Roda Rani Konadhode, Dheeraj Pelluru, Priyattam J. Shiromani

**Affiliations:** ^1^Departments of Psychiatry and Behavioral Sciences, Medical University of South CarolinaCharleston, SC, USA; ^2^Ralph H. Johnson VA Medical Center, Medical University of South CarolinaCharleston, SC, USA

**Keywords:** sleep, melanin concentrating hormone, optogenetics, hypothalamus

## Abstract

Neurons containing orexin (hypocretin), or melanin concentrating hormone (MCH) are intermingled with each other in the perifornical and lateral hypothalamus. Each is a separate and distinct neuronal population, but they project to similar target areas in the brain. Orexin has been implicated in regulating arousal since loss of orexin neurons is associated with the sleep disorder narcolepsy. Microinjections of orexin into the brain or optogenetic stimulation of orexin neurons increase waking. Orexin neurons are active in waking and quiescent in sleep, which is consistent with their role in promoting waking. On the other hand, the MCH neurons are quiet in waking but active in sleep, suggesting that they could initiate sleep. Recently, for the first time the MCH neurons were stimulated optogenetically and it increased sleep. Indeed, optogenetic activation of MCH neurons induced sleep in both mice and rats at a circadian time when they should be awake, indicating the powerful effect that MCH neurons have in suppressing the wake-promoting effect of not only orexin but also of all of the other arousal neurotransmitters. Gamma-Aminobutyric acid (GABA) is coexpressed with MCH in the MCH neurons, although MCH is also inhibitory. The inhibitory tone of the MCH neurons is opposite to the excitatory tone of the orexin neurons. We hypothesize that strength in activity of each determines wake vs. sleep.

## Early history of sleep research

In the last 100 years significant progress has been made in identifying the neurons that wake us up and make us fall asleep. The history behind the research effort is quite fascinating and involves the influenza epidemic of 1918. It was during that epidemic that a young Viennese physician named Baron von Economo concluded that sleep and waking were generated from specific areas of the brain (Economo, 1930). von Economo’s conclusions were revolutionary since at that time it was believed that sleep occurred because people simply closed their eyes. Von Economo performed autopsies on some of the patients who had succumbed to the disease and from his observations he concluded that there was a region in the rostral part of the hypothalamus that was responsible for sleep and a region in the posterior hypothalamus that was responsible for wake.

At that time many distinguished scientists were intrigued by sleep and wake centers in the brain and were actively involved in trying to unravel the mysteries of the sleeping brain. One such investigator was Walle Nauta who was conducting his studies during the height of the Second World War in Holland. He wanted to determine whether rats with lesions in the brain, similar to those found in humans by von Economo, could have changes in sleep and wake. He did not have an electroencephalogram to record brain activity, but solely from behavioral observations he came to the same conclusion as von Economo (Nauta, 1946). Moruzzi and Magoun then showed that a sedated animal without any lesions could be aroused by electrically stimulating the brainstem reticular core (Moruzzi and Magoun, 1949).

### Discovery of REM sleep

Everything changed in 1953 when REM sleep was discovered (Aserinsky and Kleitman, 1953). The discovery was made by Eugene Aserinsky, a graduate student in the laboratory of Nathaniel Kleitman at the University of Chicago. Aserinsky’s doctoral thesis was to describe the pattern of eye movements in infants. He connected his 8 year-old son Armond to an EEG machine and noticed that at various points in the night, the pattern of the EEG activity and the eye movements began to resemble what one would notice during waking (Brown, 2003). He rushed in to the room to see if the boy was awake and much to his surprise he was fast asleep, but yet the EEG looked as if he was awake. He could not readily explain why there would be a sudden shift in the EEG to a waking EEG even though the boy was behaviorally asleep. His mentor was equally skeptical and both felt that they were recording some sort of artifact. Aserinsky methodically eliminated all doubts and once Kleitman was convinced, they published their paper in the journal Science. It was a simple paper that described regularly occurring periods of eye movements every 90 min or so, and that these periods occurred with a waking EEG.

Their discovery was a landmark in neuroscience research because it clearly showed for the first time that every 90 min the brain awakened itself during sleep. This new sleep state was called rapid eye movement sleep or REM sleep because of the occurrence of eye movements. The other phase of sleep was called non-REM sleep. REM sleep represents a paradox in that behaviorally one can see that the person is asleep, yet the pattern of the EEG activity resembles what is seen during waking. Another name for REM sleep is “paradoxical sleep”. Indeed, it is now very clear that the activity of the brain during a REM sleep episode is similar to waking.

Very quickly other researchers began to investigate this new phase of sleep and it was discovered that it was present in lower animal species as well. By the late 1950’s a French neuroscientist, Michel Jouvet, determined that REM sleep was generated from the brainstem (Jouvet, 1962, 1972). We now know that there are neurons in the pontine brainstem that are responsible for generating REM sleep (Kaur et al., 2009). However the signal that initiates REM sleep emanates from the hypothalamus. In other words, we have now come full circle to von Economo.

## Orexin, waking and narcolepsy

In 1998 two independent groups using different approaches discovered orexin (also known as hypocretin) (de Lecea et al., 1998; Sakurai et al., 1998). The distribution of orexin-containing neurons has been plotted in the mouse, rat (de Lecea et al., 1998; Peyron et al., 1998; Sakurai et al., 1998; Nambu et al., 1999) and humans (Elias et al., 1998; Thannickal et al., 2000), and we have plotted its distribution in the cat (Wagner et al., 2000). Orexin neurons project to virtually the entire brain and spinal cord, providing especially heavy innervation to regions implicated in the regulation of wakefulness such as the tuberomammillary nucleus (TMN) and the locus coeruleus (LC; Peyron et al., 1998). There are two orexin receptors (orexin-1 and orexin-2 receptors; or hypocretin 1 and hypocretin-2 receptors) and their distribution in the brain has been determined (Greco and Shiromani, 2001; Marcus et al., 2001). These receptors are especially heavy in areas implicated in wakefulness such as the LC (mainly orexin-1 receptors), TMN (mainly orexin-2 receptors), the dorsal raphe, and the basal forebrain.

In 1999 these peptides were linked to narcolepsy, a sleep disorder characterized by excessive daytime sleepiness and sudden muscle paralysis (called cataplectic attacks). Canines with narcolepsy were found to have a mutation in the orexin-2 (hypocretin-2) receptor (Lin et al., 1999) while mice lacking the orexin peptide (Chemelli et al., 1999) or the neurons containing orexin (hypocretin) (Hara et al., 2001) displayed behavioral and EEG signs of narcolepsy. Human narcoleptics have low to negligible levels of orexin-A in the cerebrospinal fluid (CSF) (Nishino et al., 2000), indicating a defect in release of the peptide, or actual loss of the orexin neurons. That same year, examination of post-mortem tissue revealed massive loss of the orexin neurons in the brains of subjects with narcolepsy (Peyron et al., 2000; Thannickal et al., 2000). In narcolepsy the orexin neurons are likely destroyed since other markers that colocalize with orexin, such as dynorphin and NARP, are also absent in humans with narcolepsy (Blouin et al., 2005; Crocker et al., 2005).

### What kills orexin neurons in narcolepsy?

It is not clear what kills the orexin neurons. Human narcolepsy is considered to be an autoimmune disease because of its linkage with the human leukocyte antigen-DQB1^*^0602 (Mignot et al., 1995, 1997). There appears to be a link with influenza based on the evidence that the influenza epidemic of 1918 caused people to be excessively sleepy and those that died were found to have lesions in the posterior hypothalamus (Economo, 1930). Indeed, in 2009 an H1N1 influenza strain appeared and a vaccine was created to prevent a global epidemic. The vaccine contained three genes derived from H1N1 and batches were made with and without adjuvants. By 2010 there emerged a cluster of cases in Europe, especially children, who displayed sudden-onset narcolepsy. An initial report (De la Herrán-Arita et al., 2013) suggesting a possible mimicry between epitopes on endogenous orexin (hypocretin) and the influenza protein has not been borne out.

### Orexin, arousal and cataplexy

Orexin neurons discharge only during waking, especially with movement (Lee et al., 2005; Mileykovskiy et al., 2005). Thus, it is not surprising that when these neurons are lost, as in narcolepsy, patients have excessive daytime sleepiness and frequent sleep attacks. Narcoleptic patients also suddenly lose motor tone and collapse, especially in response to emotional stimuli, such as laughter or anger (Aldrich, 1991). These bouts are referred to as cataplexy. Canines with narcolepsy have a mutation in the orexin-2 (hypocretin-2) receptor and display cataplexy (Lin et al., 1999). The orexin control of muscle tone is through a specific pathway in the pons and medulla (Peever et al., 2014). Mice that lack orexin (Chemelli et al., 1999) or the orexin neurons (Hara et al., 2001) display all of the symptoms of narcolepsy. Mice that lack the orexin-1 (hypocretin-1) receptor have excessive sleepiness, albeit cataplexy is not as severe as the ligand knockouts (Willie et al., 2003). Mice with deletions of the orexin-2 receptor have a severe cataplexy as the ligand knockouts (Kalogiannis et al., 2011). We have linked orexin-B to the neurotoxin saporin to kill the orexin neurons and find that the rats display narcoleptic symptoms (Gerashchenko et al., 2001).

Because the orexin neurons are nestled with neurons that control other behaviors new genetically engineered tools are necessary to selectively manipulate only the orexin neurons. These new tools include optogenetics and DREDD (Designer Receptors Excitated by Designer Drugs). Optogenetic activation of the orexin neurons produces arousal (Adamantidis et al., 2007; Carter et al., 2010; Tsunematsu et al., 2011) and inhibition with DREDD produces sleep (Sasaki et al., 2011).

Based on the converging evidence from human narcolepsy, neuroanatomy, electrophysiology, pharmacology, knockout (ligand and receptor), and the new optogenetic/DREDD data a network model has emerged that hypothesizes that the orexin neurons regulate arousal and muscle tone by activating downstream arousal neurons in the basal forebrain, TMN, dorsal raphe, and the LC.

### Orexin gene transfer to rescue narcolepsy symptoms

We pioneered the use of gene therapy to rescue narcolepsy symptoms in mouse models of the disease. In three studies we reinserted the gene for orexin into surrogate neurons and convincingly demonstrated that it decreased cataplexy (Liu et al., 2008, 2011; Blanco-Centurion et al., 2013). We demonstrated that there is site-specificity in that the orexin gene must be inserted into surrogate neurons that are part of the circuit regulating cataplexy. Moreover, the surrogate neurons must be active during the cataplexy bout because that would release the orexin onto target neurons and stabilize the circuit (Bourgin et al., 2000; Huang et al., 2001). Another group transferred the gene for the orexin receptors into mice that lacked both the orexin receptors (Hasegawa et al., 2014). Reinsertion of the orexin-2 receptor specifically into the serotonergic dorsal raphe neurons decreased cataplexy while insertion of the orexin-1 receptor only in noradrenergic LC neurons increased waking. Such, mapping studies using genetically engineered tools are elucidating specific circuits regulating sleep and wake.

## Sleep-active neurons

Much of the research in sleep neurobiology has focused on the arousal neurons. However, what shuts-off the arousal neurons so that sleep can ensue? One possibility is that during waking endogenous factors such as adenosine, cytokines and prostaglandins accumulate and inhibit the waking neurons (Krueger et al., 2011). Glia may also release adenosine to increase sleep pressure (Halassa et al., 2009). The decrease in activity of arousal neurons allows sleep-active neurons to become active and generate sleep. These sleep-active neurons begin to fire in drowsiness and then increase their firing during non-REM sleep and REM sleep (Jones, 2011). The increase in activity of the sleep-active neurons potently shuts off the wake-active neurons to induce sleep. Sleep-active neurons have been identified in the preoptic area (Sherin et al., 1996), the cortex (Gerashchenko et al., 2008), and in the lateral hypothalamus (Hassani et al., 2009).

### The galanin-positive sleep-active neurons in the preoptic area

The preoptic area was initially identified by von Economo and Nauta as being important for sleep since discrete lesions of this region produced insomnia. It is now known that stimulation of this region by small electrical currents, warming or by pharmacological means will produce sleep (McGinty and Szymusiak, 2000). c-Fos, an immediate early gene and a marker of neuronal activity, has helped to identify the phenotype of the sleep-active neurons and define the region and the connection to arousal neurons (Sherin et al., 1996). c-Fos expressing sleep-active neurons are present in the ventral lateral preoptic area (VLPO; Sherin et al., 1996) and the median preoptic area (MnPN; Alam et al., 1995; Szymusiak et al., 1998; Gong et al., 2000; Suntsova et al., 2002). These sleep-active neurons contain Gamma-Aminobutyric acid (GABA) and galanin and are inhibitory to major arousal populations (Chou et al., 2002). The VLPO neurons are inhibited by acetylcholine, serotonin and norepinephrine (NE), but are unaffected by histamine (Gallopin et al., 2000).

Electrophysiology studies have confirmed that neurons in the VLPO and MnPN begin to fire during drowsiness and peak activity is seen during non-REM sleep. The sleep-active cells comprise about 25% of the recorded cells in the basal forebrain-preoptic area and are intermixed with wake-active cells which predominate. Thus, the activity of the sleep-active neurons would release inhibitory agents at target wake-active neurons, shutting them off and triggering sleep (McGinty and Szymusiak, 2000). Lesions of the VLPO decrease sleep and increase wake (Lu et al., 2000), thereby replicating Nauta’s and von Economo’s observations. When the lesions extend dorsally then REM sleep is decreased, suggesting that this region influences pontine REM sleep generator neurons. The VLPO and MnPN neurons may become active in response to sleep pressure (Gvilia et al., 2006a,b). The preoptic area sleep-active neurons in the VLPO and MnPN may convey light-dark information since they receive input from the retina (Lu et al., 1999). Since the sleep-active neurons are in a minority and are nestled with neurons regulating waking it is important to selectively stimulate phenotypically identified GABA/galanin sleep-active neurons. This will provide direct evidence linking them to sleep generation. The new methods, such as optogenetics and DREDD, enable selective manipulation of neurons, which is an advantage over non-selective neuronal activation with electrical studies.

### Neurons containing melanin concentrating hormone (MCH)

The sleep-active neurons in the preoptic area (VLPO and MnPN) are still considered to be the only neurons responsible for sleep in current models of sleep-wake regulation (Saper et al., 2010).

However, sleep-active neurons outside the preoptic area have been found (Jones, 2011). Some of these neurons contain melanin concentrating hormone (MCH; Hassani et al., 2009). In head restrained rats MCH neurons are quiet during waking, begin firing during non-REM and are most active during REM sleep (Hassani et al., 2009).

Mammalian MCH is a 19 amino acid hormone synthesized as a prepro-hormone encoding two additional peptides neuropeptide EI and neuropeptide GE (NGE) which are cleaved by post-translational modification (Nahon, 1994). MCH neurons co-express GABA (Elias et al., 2008), CART (cocaine- and amphetamine-regulated transcript; Broberger, 1999), and nesfatin (Fort et al., 2008).

**Figure 1:**
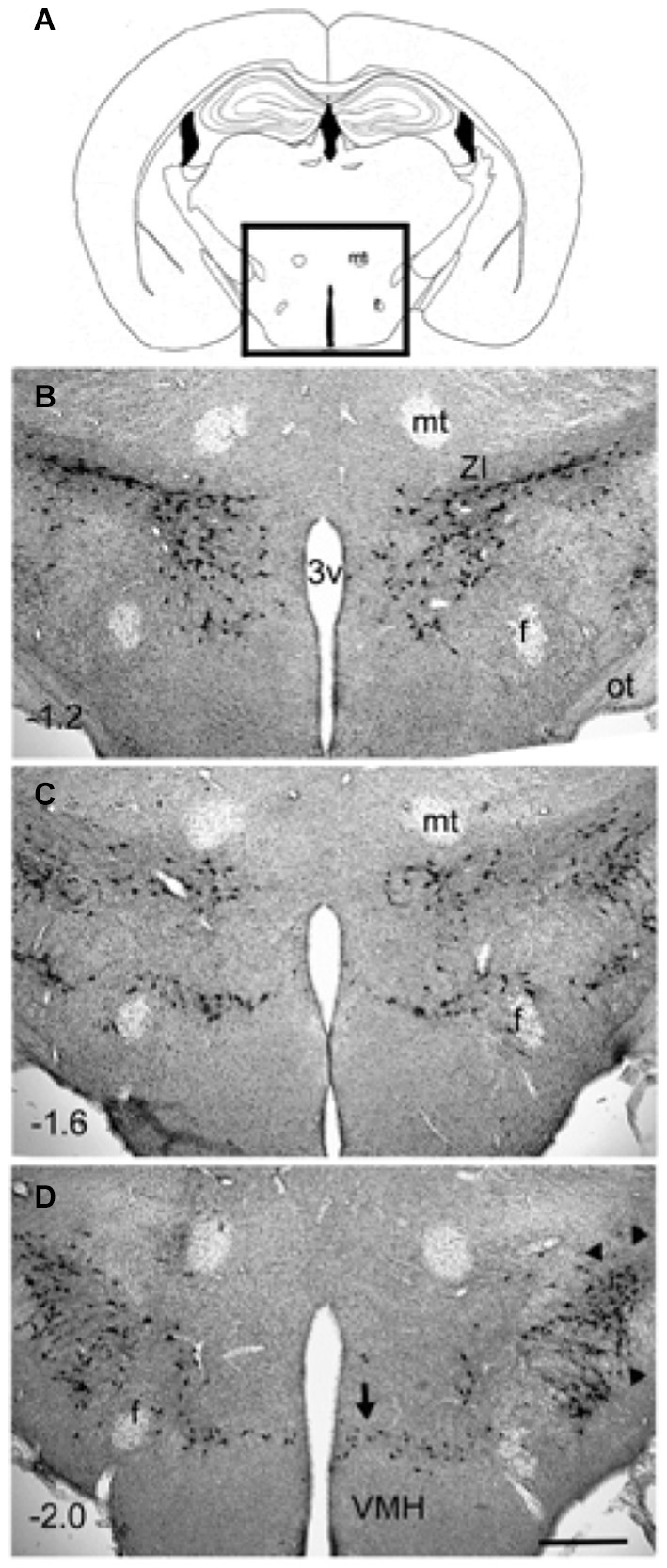
**Distribution of MCH-immunoreactive neurons in wildtype C57Bl6/j mice (3 months old)**. Coronal sections (40 µm thick) were processed for immunohistochemical detection of MCH-immunoreactivity (rabbit anti-MCH; 1:5000 dilution; overnight incubation) and visualized using the avidin-biotin -diaminobenzidene method. The MCH-ir neurons were present only in the hypothalamus (boxed area in photo **A**). A major cluster is located in the zona incerta (photo **B**) and extends medially to the ventricle. Another major cluster is located laterally (arrowheads in **D**). Minor clusters are located around the fornix (photo **C**) and ventrally along the dorsal border of the VMH (arrow in photo **D**). The numbers in **(B–D)** represent distance (millimeters) caudal to bregma. The calibration bar in **(D)** = 250 µm. Abbreviations: 3v= third ventricle; f = fornix; mt = mammillothalamic tract; ot = optic tract; ZI = zona incerta.

However, GABA, nesfatin and CART are also found in non-MCH neurons. MCH expressing neurons are present in the zona incerta, dorsomedial hypothamus and lateral hypothalamus (Elias et al., 1998; See Figure 1). Overall, MCH neurons project to the same targets as orexin neurons (Bittencourt and Elias, 1993, 1998; Elias and Bittencourt, 1997; Elias et al., 2008). MCH neurons have been shown by *in situ* hybridization, to co-localize with nociceptin/orphanin FQ opioid receptor (NOP), MCHR1, both orexin receptors (ORX), somatostatin receptors 1 and 2 (SSTR1, SSTR2), kisspeptin receptor (KissR1), neurotensin receptor 1 (NTSR1), neuropeptide S receptor (NPSR), cholecystokinin receptor A (CCKAR), and the κ-opioid receptor (Parks et al., 2014b).

There are two MCH receptors, but only MCH receptor-1 is present in rodents (Chambers et al., 1999; Tan et al., 2002). MCHR1 is expressed in hippocampus, subiculum, basolateral amygdala, shell of the nucleus accumbens, hypothalamus (ventromedial nucleus, arcuate nucleus, and zona incerta), TMN, dorsolateral pons, including the dorsal raphe, and LC (Saito et al., 2001). MCH couples to the Gi alpha subunit and inhibits production of cAMP (Saito et al., 2001).

MCH knockout mice are awake and more active, have less non-REM, and are lean (Zhou et al., 2005; Willie et al., 2008). MCHR1 knockout mice have overall normal sleep levels and a normal sleep rebound after sleep deprivation (Adamantidis et al., 2008). Transgenic mice overexpressing prepro-MCH are hyperphagic and develop mild obesity with insulin-resistance (Ludwig et al., 2001). Ablation of the MCH neurons (MCH-ataxin; 60–70% loss) results in mice that are lean, hypophagic, having increased temperature and energy expenditure (Alon and Friedman, 2006), but sleep has not been recorded in these mice.

#### Effects of MCH on sleep

Intracerebroventricular (ICV) injection of MCH during the dark period dose dependently increases REM sleep by 200% and non-REM by 70% (Verret et al., 2003). MCH injection into sleep promoting areas such as VLPO significantly increase non-REM (Benedetto et al., 2013) while injection into REM sleep areas such as nucleus pontis oralis or dorsal raphe increase REM sleep (Torterolo et al., 2009; Lagos et al., 2011). Luppi’s group has suggested that MCH neurons regulate REM sleep based on c-Fos expression in MCH neurons after REM sleep deprivation (Verret et al., 2003; Hanriot et al., 2007).

We have measured CSF levels of MCH in rats and find that it is highest during the day when the rats have the most sleep (Pelluru et al., 2013). In the same rats, orexin levels were highest at night, which is when the rats are awake. In humans, MCH is also associated with sleep and orexin with waking (Blouin et al., 2013). Thus, orexin and MCH levels are reciprocal to each other just like their firing pattern. They also have opposite effect on sleep-wake.

#### Pharmacology of MCH neurons (partial list; Figure 3)

**Figure 2:**
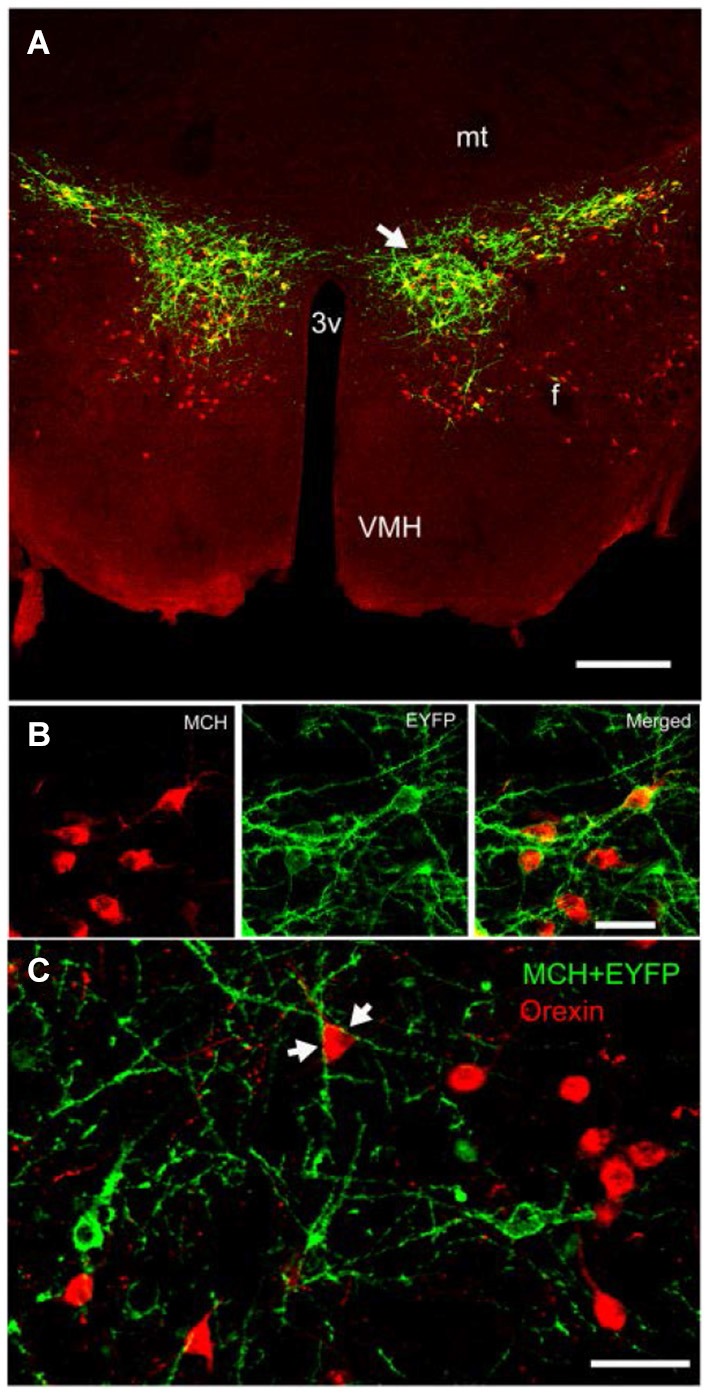
**Expression of ChR2-eYFP in MCH-immunoreactive neurons in the lateral hypothalamus of a representative wildtype C57BL/6J mouse**. Compare with Figure 1. Panel **(A)** depicts the expression of ChR2-eYFP (green) in MCH neurons (red). Notice that the MCH neurons are diffusely distributed around the fornix and many MCH neurons located medial and ventral to the fornix did not contain the light-sensitive opsin. Panel **(B)** is a higher magnification view of a cluster of MCH neurons (arrow in panel **A**) that also contain ChR2-EYFP. Panel **(C)** depicts the close relationship between the orexin neurons (red) and MCH neurons (green ChR2-eYFP). The arrows in panel **(C)** show the encirclement of an orexin soma by MCH-ChR2-EYFP processes. Scale bar in **(A)** is 250 µm, **(B)** is 50 µm and **(C)** is 60 µm. Abbreviations: 3V = third ventricle; mt = mammillothalamic tract; mfb = medial forebrain bundle; f = fornix; VMH = ventromedial hypothalamus.

**Figure 3:**
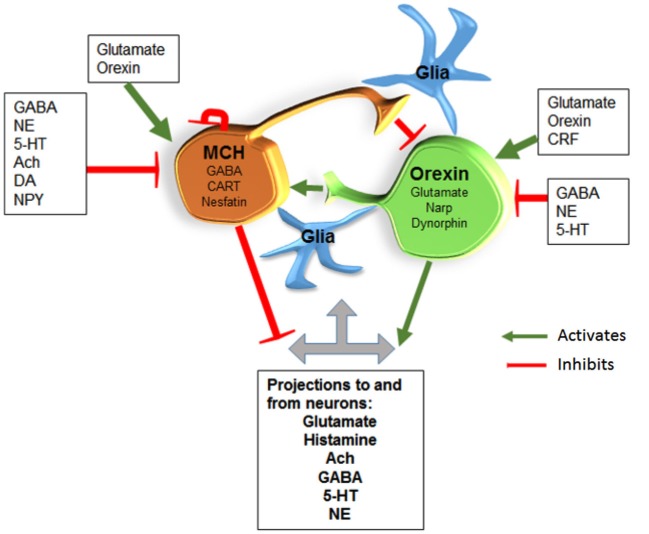
**MCH/GABA neurons are antagonistic to the orexin neurons**. We hypothesize that local interaction between these two opposing neurons regulate sleep and wake. Glia are also likely to interact with neurons along the lines hypothesized by Haydon’s studies. Abbreviations: Gamma-Aminobutyric acid (GABA); Norepinephrine (NE); Serotonin (5-HT); Acetylcholine (Ach); Dopamine (DA); Neuropeptide Y (NPY); Corticotropin releasing factor (CRF); Cocaine-Amphetamine-Regulated Transcript (CART); Neuronal activity regulated pentraxin (Narp).

MCH neurons are excited by orexin directly, and indirectly by enhancing glutamate release from excitatory neurons synapsing on MCH neurons (van den Pol et al., 2004; Huang and van den Pol, 2007). MCH neurons are excited by AMPA agonists and NMDA (van den Pol et al., 2004; Huang and van den Pol, 2007). MCH neurons are also excited by cannabinoid type-1 receptor (CB1R) agonist (Huang et al., 2007). This might explain the sleep-inducing effects of marijuana (Huang et al., 2007).

The peptide MCH inhibits orexin neurons, an effect not seen in MCHR1KO (Rao et al., 2008). MCH also inhibits neighboring GABA neurons (Gao and van den Pol, 2001). MCH neurons are inhibited by MCH, GABA, NE (effect mediated by alpha-2 receptor), serotonin, acetylcholine (muscarinic), Neuropeptide Y (NPY; Gao et al., 2003; van den Pol et al., 2004) and Histamine (Parks et al., 2014a). Dopamine (DA) inhibits MCH neurons through alpha-2 receptor (Alberto et al., 2011) and also D1 and D2 like receptors (Conductier et al., 2011).

#### Optogenetic activation of MCH neurons

The MCH neurons are virtually silent during waking, increase their discharge rates during sleep and reach their peak activity during REM sleep (Hassani et al., 2009). Based on their activity patterns we hypothesized that activating the neurons during waking should hasten sleep onset. To test this hypothesis we inserted the gene for the light-sensitive cation channel, channelrhodopsin-2, into MCH neurons in wildtype C57Bl6/j mice (Figure 2). We chose not to use the MCH-Cre mice, which have limited testing the hypothesis only in mice. Instead, we created our own MCH-promoter driven vector (supplied by Anthony van den Pol) to insert the ChR2+EYFP into MCH neurons in all vertebrate species. Three weeks after gene insertion, we recorded sleep for 48 h and then stimulated the MCH neurons with 473 nm blue light pulses (10 ms; 1 min on every 5 min; Konadhode et al., 2013). The stimulation began at lights-off, which is the start of the active phase of nocturnal rodents. We found that such stimulation reduced the length of waking bouts and increased both non-REM and REM sleep. The increase in sleep was most robust when the lights pulses were given at 10 Hz compared to 0 Hz or 5 Hz. The 10 Hz stimulation also increased delta power, a marker of sleep intensity. We did not find that optogenetic activation of MCH neurons during the light phase, which is the rest phase of nocturnal rodents, had any effect on sleep. We concluded that because the mice are normally asleep during the day it is not possible to generate more sleep. In other words, during the light phase there is a ceiling effect.

Another group (Jego et al., 2013) used MCH-Cre mice, and used a different stimulation paradigm compared to ours. They stimulated only during the second half of the light phase and only once the mice entered into REM sleep. They found that such stimulation during REM sleep prolonged the REM sleep bout. They also used light-sensitive halorhodopsin, eNpHR3.0, to inhibit the MCH neurons, but it did not abort REM sleep bouts. They did not stimulate during waking or at night.

A third group expressed the gene for diphtheria toxin in the MCH neurons to selectively kill the MCH neurons (Tsunematsu et al., 2014). When the MCH neurons were ablated the mice were awake more during the day and night and had a selective decrease in non-REM sleep, but no change in amount of REM sleep. That study also found that optogenetic stimulation of the MCH neurons at night decreased the length of waking bouts at night and increased the number of non-REM sleep bouts (Figures 2A,B in Tsunematsu et al., 2014). In their study inhibition of the MCH neurons during the day had no effect on sleep, which confirms what was found by Jego et al. (2013).

The difference between levels of non-REM and REM sleep between the three studies might be related to activation of specific population of MCH neurons. As seen in Figure 1 the MCH neurons extend about 1 mm along the anterior posterior plane, and are diffusely distributed about 1.5 mm laterally from the midline, and about 1 mm along the dorsal-ventral plane. MCH neurons are located densely in the zona incerta, in the lateral portions of the lateral hypothalamus, along the perifornical area, and ventrally along dorsal ridge of the ventromedial hypothalamus. In our study ChR2 was robustly expressed in the MCH neurons in the zona incerta, and less so in the lateral divisions (see Figures 1, 2). Moreover, in our study, we stimulated about half of the total population of MCH neurons, and found robust increases in both non-REM and REM sleep. In particular, the increase in sleep occurred against a strong waking drive indicating that MCH neurons can suppress the combined activity of all of the arousal neurons. The other two studies infected about 88% of the MCH neurons but given the diffuse distribution of the MCH neurons, and the fact that mammalian brain tissue heavily scatters light so that only about 10% of light reaches to about 500 µm (Adamantidis et al., 2007; Aravanis et al., 2007), it is very difficult to reliably stimulate all MCH+ChR2-positive neurons with optogenetics. Nevertheless, these three studies underscore the importance of MCH neurons in both non-REM and REM sleep. It is likely that a pharmacogenetic approach with DREDD may reach all the MCH neurons. However, the drawback of DREDD is that it is short-lived (because of half-life of the drug), and does not provide millisecond control of targeted neurons.

The MCH peptide is inhibitory and inhibits orexin neurons (Rao et al., 2008). It also inhibits neighboring GABA neurons (Gao and van den Pol, 2001). MCH neurons also co-express GABA. Thus, both MCH and GABA are likely to inhibit target neurons. Thus, it is not surprising that activation of MCH neurons is able to shut-off the arousal neurons and induce sleep. When they are lost, as in the Tsunematsu et al. (2014) study, then the orexin neurons are likely to be more active resulting in increasing waking.

MCH has been implicated in feeding but optogenetic activation of MCH neurons induced sleep, not feeding (Jego et al., 2013; Konadhode et al., 2013). In our study, we found that mice slept rather than eat at a circadian time point when they normally should be eating (Konadhode et al., 2013). In our study, the mice had satiated their sleep need and undoubtedly were hungry. Nevertheless, they slept in response to MCH neuron stimulation. However, normal sucrose preference was reversed to sucralose in response to 20 Hz optogenetic stimulation of MCH neurons (Domingos et al., 2013).

#### Theoretical framework for the regulation of wake, non-REM and REM sleep

Based on data from optogenetics, c-Fos, electrophysiology, and lesion studies we hypothesize that sleep begins with activity of the sleep-active neurons (preoptic and MCH). The MCH neurons prevent the activation of the local orexin neurons which likely decreases orexin’s drive of downstream arousal neurons. The preoptic sleep-active neurons are also inhibiting the arousal neurons. REM sleep occurs when the sleep-active neurons inhibit GABA neurons in the pons (see our model for REM sleep in Kaur et al., 2009) which then allows REM sleep-active neurons to fire and REM sleep ensues. We hypothesize that sleep ends because the MCH neurons are self-inhibiting and the wake state ends when the orexin neurons activate the MCH neurons (see Figure 3). There is support for this possibility because we find that with MCH stimulation length of wake bouts is cut in half but the length of non-REM or REM sleep bouts is unchanged. We suggest that this is because with progressive activity MCH neurons shut-off (self-inhibiting) and non-REM sleep ends. The interaction between the MCH and orexin neurons still needs to be investigated, but it provides a heuristic model of sleep-wake regulation.

Our position based on existing data is that the MCH neurons are a separate sleep promoting group that *partners* with the preoptic sleep-active neurons in generating sleep. Can sleep occur without the MCH neurons? Yes, since the preoptic area sleep-active neurons are intact. Which dominates: preoptic or MCH neurons? We think that each influences sleep based on their input. For instance, preoptic sleep-active neurons receive direct input from retina (Lu et al., 1999), are entrained to the light-dark cycle and could control timing of sleep. MCH neurons are within an area serving energy metabolism and respond to glucose (Burdakov et al., 2005). Both the MCH and the hypocretin/orexin neurons are located in a region sensing energy metabolism. A rise in glucose activates MCH neurons (Burdakov et al., 2005; Kong et al., 2010), which may explain post-prandial sleep.

## Conclusions

More work needs to be done to fully identify the interaction between the sleep-active and arousal neurons in regulating sleep and wake. Important questions remain. For instance: How does the suprachiasmatic nucleus regulate these neurons? What is the relationship of energy metabolism and sleep-wake neurons? It is also important to mechanistically demonstrate that turning on sleep-active neurons shuts-off the wake-active neurons. This can now be done with optogenetics, a powerful tool that allows the neuroscientist to selectively manipulate specific neurons.

## Conflict of interest statement

The authors declare that the research was conducted in the absence of any commercial or financial relationships that could be construed as a potential conflict of interest.
